# In this issue

**Published:** 2022-12

**Figure:**
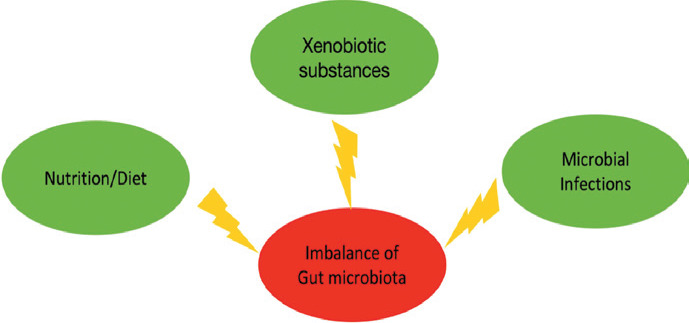
Imbalance of gut microbiota via diet, infections, nutrition, and xenobiotic.

**Effect of gut microbiota on colorectal cancer progression and treatment**

Alasiri discusses an in depth role of gut microbiota and its functions to explore the link between development of colorectal cancer (CRC) in patients and their responses to treatment. Several studies have shown that gut microbiota can induce resistance against pathogens and regulate the immune system. Colorectal cancer is the third-deadliest cancer worldwide, accounting for approximately 900,000 deaths per year globally. Gut microbiota has been heavily linked to CRC incidence and prevention via bacterial metabolites, invasion, translocation, host’s defense modulations, and bacterial-immune system interactions. In addition, it can influence the metabolism of chemical compounds such as drugs and xenobiotics to manipulate the treatment response in CRC patients.

*see page 1289*

