# Supplementary material for: In this issue
Source: Saudi Med J. 2022 Dec;43(12):1287. (PMC9994519)
Supplement: Related texts

# Abstract: Original Articles

**Investigation of preoxygenation methods in cesarean surgeries with the oxygen reserve index**

Kocakulak et al investigate preoxygenation methods that were carried out for 3 minutes (min) at tidal volume and 30 seconds (s) with the 4 deep vital capacity technique using the Oxygen Reserve Index (ORI) among pregnant women. The patients were randomly divided into 2 groups with the provision of preoxygenation using 100% O_2_ at a rate of 10 L.min-1 for 3 min at normal tidal volume (Group 1) and 30 s with the 4 deep vital capacity technique (Group 2). The study was completed with 66 patients. Fraction of inspired oxygen (FiO_2_) values were found to be low in T1 (*p*=0.012) in Group 1, and high in fraction of expired (FeO_2_) values in T1 and T2 (*p*=0.025 and *p*=0.009) in Group 2, while no significant differences were found at other times (*p*>0.05). We obtained greater FiO_2_ and FeO_2_ values in preoxygenation with the 30 s 4 deep vital capacity method, and because this method did not cause a significant decrease in the post-intubation ORI values, we believe that the usage of this method in cesarean section surgeries may be appropriate.

***see page 1317***

**The role of laparoscopy in emergency colorectal surgery**

Alselaim et al assess the outcomes of the laparoscopic approach compared to those of the open approach in emergency colorectal surgery 241 patients (182 open resections, 59 laparoscopic approaches) were included in this study. Thee length of stay in the intensive care unit was shorter in the laparoscopic than in the open group (1±3 days verses 7±16 days). After multivariable logistic regression, patients undergoing laparoscopic resection had a 70% lower risk of surgical site infection than those undergoing open surgery (adjusted odds ratio=0.33, 95% confidence interval: [0.06-1.67]), a difference that was not significant. The use of laparoscopy in emergency colorectal surgery is safe and feasible, with a trend toward better outcomes. Colorectal surgery specialization is an independent predictor of an increased likelihood of undergoing laparoscopy in emergency colorectal surgery.

***see page 1333***

**Prevalence of transfusion-transmitted infections in Saudi Arabia blood donors. *A nationwide, cross-sectional study***

Alsughayyir et al establish a nationwide epidemiological profile of transfusion-transmittable infection (TTI) markers among seemingly healthy blood donors to update policies required to ensure blood safety. A total of 375,218 whole blood units were donated. Notably, the central and southern regions were the epicenters of TTI-reactive blood donors. Hepatitis B virus (HBV) markers accounted for 85.7% and were the overall most prevalent of TTI-positive donations, followed by hepatitis C virus (HCV) at 5.9% and syphilis at 5.6%. In particular, anti-HBc and hepatitis B surface antigen (HBsAg) were most prevalent in the south, while HBV NAT was highest in the center. Hepatitis B virus, HCV, and syphilis carry the greatest risk of TTI in Saudi Arabia. Including HBsAg screening is a necessary precautious measure to maintain blood safety.

*see page 1363*

**Figure:** Map of Saudi Arabia demonstrating the prevalence of transfusion-transmittable infection-positive markers to total confirmed cases in each geographical area.
